# Protective Effects of Low-Dose Alcohol against Acute Stress-Induced Renal Injury in Rats: Involvement of CYP4A/20-HETE and LTB_4_/BLT1 Pathways

**DOI:** 10.1155/2021/4475968

**Published:** 2021-10-13

**Authors:** Yongping Chen, Haotian Yang, Tianyuan Yang, Haiyang Zhang, Yuan Zhao, Lin Li, Honggang Fan

**Affiliations:** College of Veterinary Medicine, Northeast Agricultural University, Harbin 150030, China

## Abstract

Low-dose alcohol possesses multiple bioactivities. Accordingly, we investigated the protective effect and related molecular mechanism of low-dose alcohol against acute stress- (AS-) induced renal injury. Herein, exhaustive swimming for 15 min combined with restraint stress for 3 h was performed to establish a rat acute stress model, which was verified by an open field test. Evaluation of renal function (blood creatinine and urea nitrogen), urine test (urine leukocyte esterase and urine occult blood), renal histopathology, oxidative stress, inflammation, and apoptosis was performed. The key indicators of the cytochrome P450 (CYP) 4A1/20-hydroxystilbenetetraenoic acid (20-HETE) pathway, cyclooxygenase (COX)/prostaglandin E_2_ (PGE_2_) pathway, and leukotriene B_4_ (LTB_4_)/leukotriene B_4_ receptor 1 (BLT1) pathway were measured by real-time PCR and ELISA. We found that low-dose alcohol (0.05 g/kg, i.p.) ameliorated AS-induced renal dysfunction and histological damage. Low-dose alcohol also attenuated AS-induced oxidative stress and inflammation, presenting as reduced malondialdehyde and hydrogen peroxide formation, increased superoxide dismutase and glutathione activity, and decreased myeloperoxidase, interleukin-6, interleukin-1*β*, and monocyte chemoattractant protein-1 levels (*P* < 0.05). Moreover, low-dose alcohol alleviated AS-induced apoptosis by downregulating Bax and cleaved caspase 3 protein expression and reduced numbers of terminal deoxynucleotidyl transferase-mediated dUTP nick-end label-positive cells (*P* < 0.01). Correlation analysis indicated that 20-HETE was strongly correlated with oxidative stress, while LTB_4_ was strongly correlated with inflammation. Low-dose alcohol inhibited AS-induced increases in CYP4A1, CYP4A2, CYP4A3, CYP4A8, and BLT1 mRNA levels and LTB_4_ and 20-HETE content (*P* < 0.01). Interestingly, low-dose alcohol had no effect on COX1 or COX2 mRNA expression or the concentration of PGE_2_. Furthermore, low-dose alcohol reduced calcium-independent phospholipase A_2_ mRNA expression, but did not affect secreted phospholipase A_2_ or cytosolic phospholipase A_2_ mRNA expression. Together, these results indicate that low-dose alcohol ameliorated AS-induced renal injury by inhibiting CYP4A/20-HETE and LTB_4_/BLT1 pathways, but not the COX/PGE_2_ pathway.

## 1. Introduction

Stress, commonly occurring in daily life, is a triggering or aggravating factor of many diseases that seriously threaten public health [1]. Accumulating evidence indicates that acute stress (AS) is deleterious to the body's organs and systems [2, 3]. Each year, approximately 1.7 million deaths are attributed to acute injury of the kidney, one of the organs vulnerable to AS [4]. However, to date, understanding of the etiopathogenesis and effective preventive treatments for AS-induced renal injury remain limited. Hence, exploring the exact mechanism of AS-induced renal injury and development of effective preventive therapeutics is urgently needed.

A recent study implicated oxidative stress and apoptosis in AS-induced renal injury [5]. Oxidative stress occurs when there is an imbalance between antioxidant depletion and excess oxides [6]. Excess oxidation products are implicated in mitochondrial damage, which triggers apoptosis [7]. Furthermore, inflammation, which is mediated by oxidative stress, is considered a hallmark of kidney disease [8]. Extensive research suggests that the occurrence, development, and regression of renal inflammation are tightly linked to arachidonic acid (AA) metabolism [9]. In addition, the stress hormone norepinephrine induces AA release [10]. However, whether AA metabolism is involved in AS-induced renal injury is unknown.

Ethanol, a psychoactive component of alcoholic beverages, has multiple bioactivities. Numerous experimental studies have emphasized the beneficial effects of low-dose alcohol on health, including suppression of adverse cardiovascular events induced by high-fat diet [11], amelioration of ischemic stroke [12], attenuation of social anxiety in young mice [13], alleviation of high-salt-induced hypertension [14], improvement of memory loss caused by temporary seizures [15], and elevation of emotion and social bonding [16]. Moreover, low-dose alcohol has been reported to inhibit oxidative stress [17]. Low-dose alcohol has also associated with reduced of inflammatory chemokine expression [18]. Typically, low-dose alcohol has been found to inhibit the production of leukotriene B_4_ (LTB_4_) and prostaglandin D_2_ [19]. However, the effect of low-dose alcohol on AS-induced renal injury remains elusive.

Accordingly, based on the biological properties of low-dose alcohol, we explored the protective effect and specific mechanism by which low-dose alcohol affects AS-induced renal injury. This study lays a theoretical foundation and provides a new perspective for application of low-dose alcohol in the prevention and treatment of AS-induced nephropathy.

## 2. Materials and Methods

### 2.1. Experimental Animals and Grouping

Thirty-two healthy adult male Wistar rats (180–220 g body weight), provided by the Second Affiliated Hospital of Harbin Medical University (Harbin, China), were raised in the animal house of Northeast Agricultural University (Harbin, China) under standard laboratory conditions, as previously described [20, 21]. Rats were placed in individual plastic cages (four rats per cage) and had access to water and standard rodent pellet food ad libitum. After a week of acclimatization, rats were randomly divided into four groups (*n* = 8 per group): CON, CON+Alc, AS, and AS+Alc. Low-dose alcohol was prepared by diluting ethanol (99.7% *v*/*v*; Taicang Xintai Alcohol Co., Hebei, China) with 0.9% (*w*/*v*) saline solution to a concentration of 1.58% *v*/*v*. The rat AS model was established as described in previous studies [5]. In detail, the rats were forced to swim for 15 min in water at 18–20°C (immediately removed when drowning occurred). Then, the rats were restrained by a rat fixator for 3 h (only the limbs and head were exposed). The CON and CON+Alc groups were administered 0.9% (*w*/*v*) saline solution and low-dose alcohol (0.05 g/kg) by intraperitoneal (i.p.) injection, respectively. The AS and AS+Alc groups were administered 0.9% (*w*/*v*) saline solution and low-dose alcohol (0.05 g/kg) via i.p. injection 0.5 h before AS, respectively. The low-dose alcohol administration concentration was selected to be lower than the daily standard drink (National Institutes of Health regulation, 0.2 g/kg) without any adverse effects. A study suggested that low-dose ethanol (0.05 g/kg) did not induce conditioned taste aversion and conditioned place preference [22]. The injection volume of the four groups was constant at 4 mL/kg body weight. All animal operations in this study were approved by the Experimental Animal Ethics Committee of Northeast Agricultural University (SRM-11, China) and carried out in accordance with the National Institutes of Health Guide for the Care and Use of Laboratory Animals (Bethesda, MD, USA) [23].

### 2.2. Open Field Test

An open field test (OFT) was performed 0.5 h after AS to validate successful model establishment. The apparatus for OFT consisted of a lidless black rectangular wooden box (100 cm × 100 cm × 40 cm) and video camera. Each rat was placed in the central square of the box, which was divided into 25 equally sized squares. The behavior and activity of rats were recorded by a camera for 3 min. Rearing numbers were recorded by two observers blinded to the trial group. The travel pathway, average velocity, central area activity percentage, and crossing number were analyzed by Super Maze software (Shanghai, China).

### 2.3. Sample Collection

All rats were sacrificed 30 min after OFT under anesthesia with isoflurane (Yipin Pharmaceutical Co., Hebei, China). Blood, urine, and kidney tissues were quickly collected. Blood and urine samples were left for 20 min at room temperature, followed by centrifugation (3000 × *g* for 10 min) at 4°C. Serum was used to measure urea nitrogen (BUN) and creatinine (CREA) levels. Urine supernatants were used to determine the contents of urine leukocyte esterase (LEU), urine occult blood (BLD), and prostaglandin E_2_ (PGE_2_). The dissected left kidney was fixed in 10% formalin solution for hematoxylin and eosin (H&E) staining, immunohistochemistry, and terminal deoxynucleotidyl transferase-mediated dUTP nick-end labeling (TUNEL) assay. The right kidney was rapidly frozen in liquid nitrogen and stored until detection.

### 2.4. Renal Function and Urine Tests

BUN and CREA levels were quantified using the UniCel DxC Synchron Clinical System (Beckman Coulter, Fulton, CA, USA). LEU and BLD contents were determined with a urine analyzer (IDEXX Vet Lab UA, Westbrook, ME, USA).

### 2.5. Histopathological Observation and Analysis

The 10% formaldehyde-fixed renal tissues were dehydrated in gradient series of alcohol and then embedded in paraffin. Subsequently, renal tissues were sectioned (4–5 *μ*m thickness), dewaxed with a gradient series of alcohol, and stained with H&E (Wuhan Biotechnology Ltd., Wuhan, China). All sections were observed using a light microscope (TE2000; Nikon, Tokyo, Japan) at 400x magnification. Six discontinuous fields of the renal cortex and medulla were scored in each section by two observers who were blind to the experimental group, as previously described [24]. In brief, the semiquantitative evaluation of renal injury is as follows: 0, no injury; 1, mild (<10%); 2, moderate (10%–50%); 3, severe (25%–50%); and 4, very severe (>50%).

### 2.6. Determination of Oxidative Stress Indicators, Inflammatory Cytokines, and Arachidonic Acid Metabolite Levels

Oxidative stress indexes such as malondialdehyde (MDA) concentration, hydrogen peroxide (H_2_O_2_) content, superoxide dismutase (SOD) activity, and glutathione (GSH) content, as well as levels of inflammatory factors such as myeloperoxidase (MPO), interleukin-6 (IL-6), and interleukin-1*β* (IL-1*β*), were detected using corresponding kits (Nanjing Jiancheng Bioengineering Institute, Nanjing, China). Concentration of 20-hydroxystilbenetetraenoic acid (20-HETE) was determined using an enzyme-linked immunosorbent assay kit (Jianglai Industrial Limited by Share Ltd., Shanghai, China). Moreover, concentrations of PGE_2_, LTB_4_, and phospholipase A_2_ (PLA_2_) were determined by corresponding enzyme-linked immunosorbent assay kits (Nanjing Jiancheng Bioengineering Institute). All operations were performed strictly as described in the kit manufacturer's protocol. The catalog numbers of all kits are listed in Table 1.

### 2.7. Immunohistochemistry Analysis

Immunohistochemical analysis was performed as previously described [25]. Briefly, paraffin-embedded renal tissue sections were dewaxed with xylene, dehydrated with a gradient series of alcohol, incubated with H_2_O_2_, and sealed with goat serum. Subsequently, sections were incubated with primary and secondary antibodies and labeled with horseradish enzyme. DAB was used for color development. Finally, all sections were observed and photographed under a DP73 microscope (Olympus, Tokyo, Japan).

### 2.8. TUNEL Assay

Paraffin-embedded renal tissue sections were pretreated according to the TUNEL apoptosis detection kit (Roche, Basel, Switzerland) manufacturer's instructions and then wetted for 60 min with 50 *μ*L of TdT enzyme reaction solution at 37°C. After 30 min reaction with antifluorescent antibody in the dark, sections were incubated with DAB (50–100 *μ*L) working solution for 5–30 min at room temperature. All sections were captured using a fluorescence inverted microscope (TE2000, Nikon). Apoptosis rates were calculated in six noncontinuous fields of each section by ImageJ software.

### 2.9. Determination of Protein Expression

Protein expression levels of Bax, Bcl-2, and cleaved caspase 3 (Wanlei Biotechnology, Shenyang, China) in renal tissues were determined by western blot analysis. Briefly, frozen kidney tissues were lysed with radioimmunoprecipitation assay lysis buffer mixed with phenylmethylsulfonyl fluoride (Beyotime Biotechnology, Shanghai, China). After detection of total protein concentrations with a bicinchoninic acid assay kit (Beyotime Biotechnology), samples with equal volumes of protein were separated by sodium dodecyl sulfate-polyacrylamide gel electrophoresis and transferred to polyvinylidene fluoride membranes, which were incubated with primary antibodies of Bax (1 : 1000), Bcl-2 (1 : 500), and cleaved caspase 3 (1 : 1000) in Primary Antibody Dilution Buffer (Leagene Biotechnology, Beijing, China) overnight at 4°C. After washing, membranes were incubated with goat anti-rabbit secondary antibody (ZSGB-BIO, Beijing, China) at 37°C for 2 h. All protein bands were captured with Amersham Imager 600 software (GE, Boston, MA, USA) and quantified with ImageJ.

### 2.10. Determination of Gene Level

Gene expression levels of cytochrome P450 (CYP) 4A1, CYP4A2, CYP4A3, CYP4A8, cyclooxygenase 1 (COX1), cyclooxygenase 2 (COX2), leukotriene B_4_ receptor 1 (BLT1), calcium-independent phospholipase A_2_ (iPLA_2_), secreted phospholipase A_2_ (sPLA_2_), and cytosolic phospholipase A_2_ (cPLA_2_) in renal tissues were determined with real-time PCR analysis, as previously described [26]. All primers (Table 2) were synthesized by Shanghai Bioengineering Co. (Shanghai, China). GAPDH mRNA expression levels were used as a reference to quantify relative expression levels of genes. Gene levels were quantified according to the 2^-*ΔΔ*Ct^ method.

### 2.11. Statistical Analysis

All data represent the mean ± SEM and were analyzed using IBM SPSS Statistics 23 software (Armonk, NY, USA). Statistical analysis was conducted via one-way ANOVA, followed by Tukey's post hoc test. Mean integral optical density was calculated by Image-Pro Plus software (Media Cybernetics, Bethesda, MD, USA). Correlation analyses were performed using Canoco for Windows 4.5 for Redundancy Analysis (Microcomputer Power, Ithaca, NY, USA). Values of *P* < 0.05 were considered statistically significant, and values of *P* < 0.01 were considered extremely significant.

## 3. Results

### 3.1. Validation of Acute Stress Model

**Figure 1:**
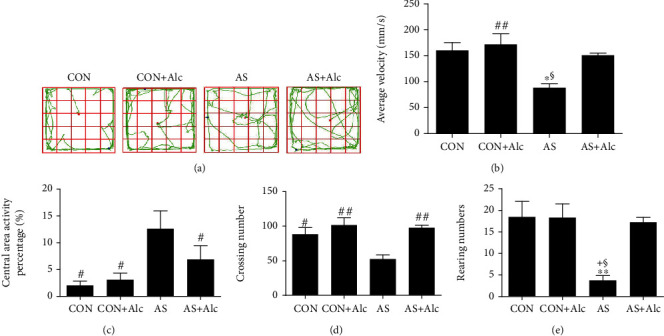
Validation of acute stress model. (a) The travel pathway of rats in OFT. (b) Average velocity of rats in OFT. (c) Central area activity percentage of rats in OFT. (d) Crossing numbers of rats in OFT. (e) Rearing numbers of rats in OFT. Data are expressed as mean ± SEM (*n* = 8). ^∗^*P* < 0.05 and ^∗∗^*P* < 0.01 versus the CON group. ^†^*P* < 0.05 versus the CON+Alc group. ^#^*P* < 0.05 and ^##^*P* < 0.01 versus the AS group. ^§^*P* < 0.05 versus the AS+Alc group. OFT: open field test; CON: control; AS: acute stress; Alc: alcohol.

To verify whether the AS model was successfully established, rats in each group underwent OFT. As show in Figure 1(a), AS rats exhibited more travel pathways in the central area and were less interested in exploring their surroundings. Average velocity (Figure 1(b)) was significantly reduced in the AS group compared with the CON (*P* < 0.05), CON+Alc (*P* < 0.01), and AS+Alc (*P* < 0.05) groups. Conversely, we observed an obvious elevation of central area activity percentage in the AS group compared with the CON, CON+Alc, and AS+Alc groups (Figure 1(c), *P* < 0.05). Moreover, the crossing numbers (Figure 1(d), *P* < 0.05) and rearing numbers (Figure 1(e), *P* < 0.01) were significantly lower in the AS group compared with the CON group. None of the results indicated significant differences between the CON and CON+Alc groups. Together, these results indicate that the AS model was successfully established.

### 3.2. Effect of Low-Dose Alcohol on Blood and Urine Indexes

**Figure 2:**
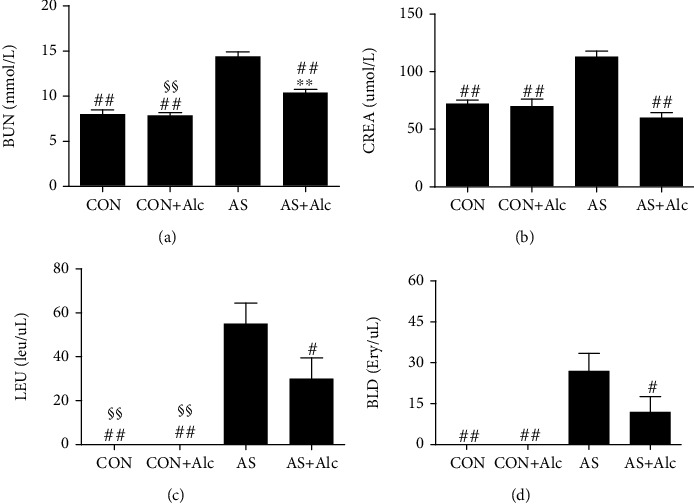
Effect of low-dose alcohol on blood and urine indexes. (a) BUN, (b) CREA, (c) LEU, and (d) BLD levels were determined. Data are expressed mean ± SEM (*n* = 5). ^∗∗^*P* < 0.01 versus the CON group. ^#^*P* < 0.05 and ^##^*P* < 0.01 versus the AS group. ^§§^*P* < 0.01 versus the AS+Alc group. BUN: urea nitrogen; CREA: creatinine; LEU: urine leukocyte esterase; BLD: urine occult blood; CON: control; AS: acute stress; Alc: alcohol.

BUN and CREA are intuitional biomarkers to evaluate renal function. LEU and BLD were measured to assess kidney injury and nephritis, respectively. As shown in Figure 2, the levels of BUN, CREA, LEU, and BLD in the AS group were remarkably increased compared with those in the CON group (*P* < 0.01), while low-dose alcohol significantly reversed the effects of AS.

### 3.3. Effect of Low-Dose Alcohol on AS-Induced Renal Histopathological Changes

**Figure 3:**
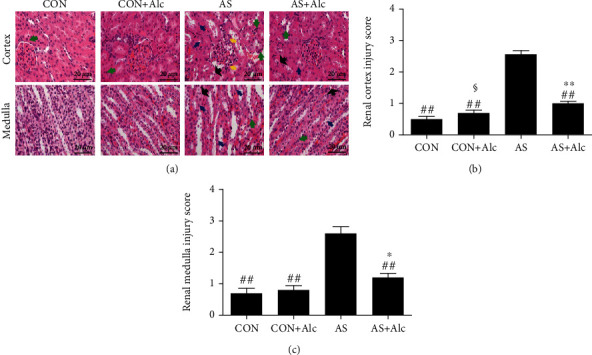
Effect of low-dose alcohol on AS-induced renal histopathological changes. (a) Representative paraffin sections of the renal cortex and medulla stained with H&E (×400); scale bar = 20 *μ*m. Black arrows indicate vacuolar degeneration. Yellow arrows indicate neutrophil infiltration. Blue arrows indicate necrotic cells. Green arrows indicate apoptotic cells. (b) Renal cortex injury score. (c) Renal medulla injury score. Data are expressed as mean ± SEM (*n* = 5). ^∗^*P* < 0.05 and ^∗∗^*P* < 0.01 versus the CON group. ^##^*P* < 0.01 versus the AS group. ^§^*P* < 0.05 versus the AS+Alc group. H&E: hematoxylin and eosin; CON: control; AS: acute stress; Alc: alcohol.

Histopathological observation was performed to visualize renal tissue injury. As shown in Figure 3(a), H&E-stained paraffin sections of the CON and CON+Alc groups showed normal renal cortex and medulla structures. In contrast, numerous vacuolated renal cells, necrotic cells, apoptotic cells, and infiltrating inflammatory cells were observed in the renal cortex and medulla of the AS group. However, low-dose alcohol significantly attenuated these renal histopathological changes induced by AS (*P* < 0.01, Figures 3(b) and 3(c)).

### 3.4. Effects of Low-Dose Alcohol on AS-Induced Oxidative Stress

**Figure 4:**
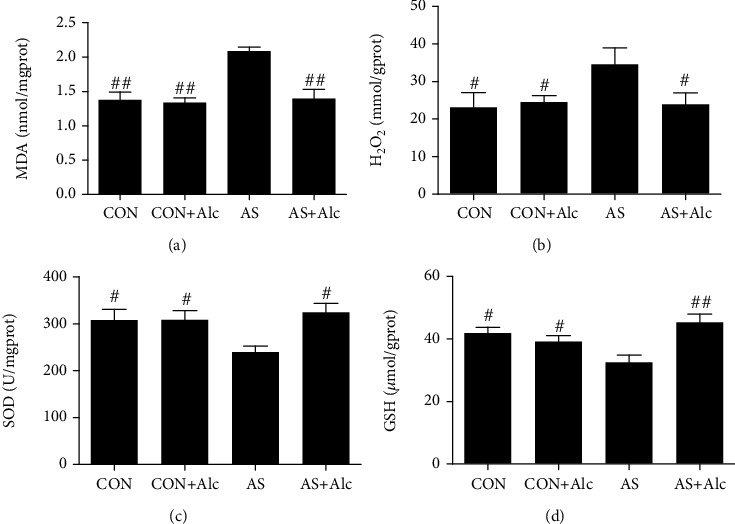
Effects of low-dose alcohol on AS-induced oxidative stress. (a) MDA level, (b) H_2_O_2_ content, (c) SOD activity, and (d) GSH concentration in rat kidney tissue were detected. Data are expressed as mean ± SEM (*n* = 6). ^#^*P* < 0.05 versus the AS group. MDA: malondialdehyde; H_2_O_2_: hydrogen peroxide; SOD: superoxide dismutase; GSH: glutathione; AS: acute stress.

Figure 4 shows that low-dose alcohol notably suppressed AS-induced overproduction of MDA (*P* < 0.01, Figure 4(a)) and H_2_O_2_ (*P* < 0.05, Figure 4(b)). In addition, SOD activity (*P* < 0.05, Figure 4(c)) and GSH concentrations (*P* < 0.01, Figure 4(d)) in the AS+Alc group were obviously elevated compared with those in the AS group.

### 3.5. Effects of Low-Dose Alcohol on MPO, Proinflammatory Cytokine, and MCP-1 Levels

**Figure 5:**
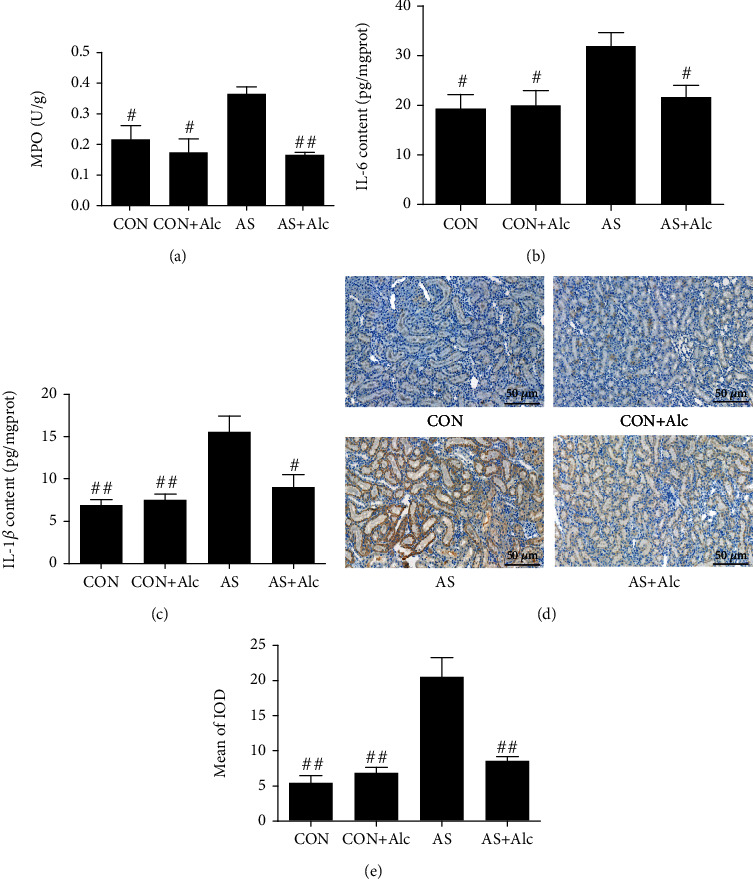
Effects of low-dose alcohol on MPO, proinflammatory cytokine, and MCP-1 levels. (a) MPO activity. (b) IL-6 content. (c) IL-1*β* content. (d) Immunohistochemistry of MCP-1 protein (×200), scale bars = 50 *μ*m. (e) Mean integral optical density (IOD) of MCP-1. Data are expressed as mean ± SEM (*n* = 6). ^#^*P* < 0.05 and ^##^*P* < 0.01 versus the AS group. MPO: myeloperoxidase; MCP-1: monocyte chemoattractant protein-1; IL-6: interleukin-6; IL-1*β*: interleukin-1*β*; AS: acute stress.

Low-dose alcohol markedly decreased MPO activity (Figure 5(a)), contents of IL-6 and IL-1*β* (Figures 5(b) and 5(c)), and levels of monocyte chemoattractant protein-1 (MCP-1) (Figures 5(d) and 5(e)), which were apparently increased in the AS group. There was no significant difference in the aforementioned changes between the CON and CON+Alc groups.

### 3.6. Effects of Low-Dose Alcohol on AS-Induced Apoptosis in the Kidney

**Figure 6:**
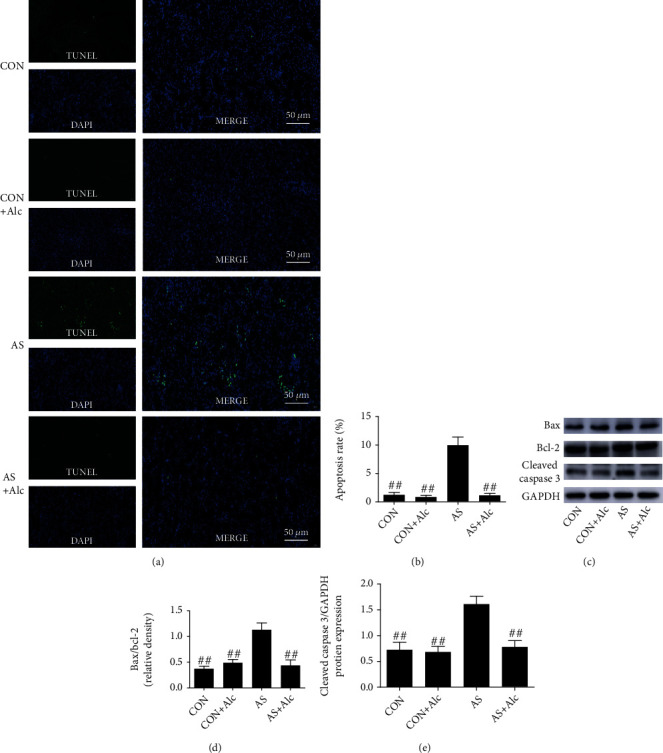
Effects of low-dose alcohol on AS-induced apoptotic in the kidney. (a) Representative TUNEL image (×200), scale bars = 50 *μ*m. (b) Apoptosis rate. (c) Western blot bands of Bax, Bcl-2, and cleaved caspase 3 proteins. (d) Bax/Bcl-2 protein expression ratios. (e) Cleaved caspase 3/GAPDH protein expression ratios. Data are expressed as mean ± SEM (*n* = 3). ^##^*P* < 0.01 versus the AS group. AS: acute stress; TUNEL: terminal deoxynucleotidyl transferase mediated nick-end labeling.

To illuminate the effect of low-dose alcohol on AS-induced apoptosis in the kidney, TUNEL staining was employed to measure apoptotic cells. Compared with the CON and CON+Alc groups, TUNEL-positive cells and percentages of apoptotic cells in the AS group were significantly increased (*P* < 0.01, Figures 6(a) and 6(b)). Moreover, the protein expression of Bax/Bcl-2 and cleaved caspase 3 was markedly higher in the AS group compared with the CON and CON+Alc groups (*P* < 0.01, Figures 6(c)–6(e)). Nevertheless, low-dose alcohol effectively blocked these AS-induced changes (*P* < 0.01).

### 3.7. Effects of Low-Dose Alcohol on the CYP4A/20-HETE Metabolic Pathway

**Figure 7:**
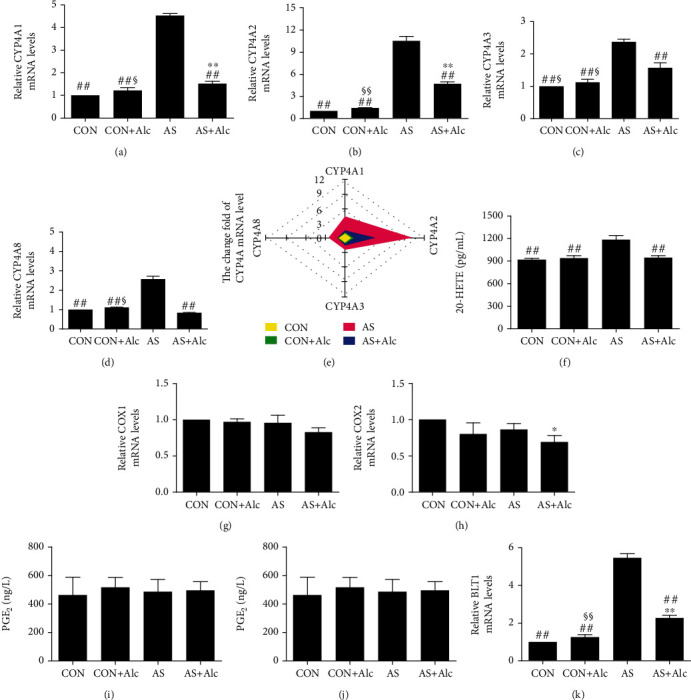
Effect of low-dose alcohol on arachidonic acid metabolism. (a) CYP4A1, (b) CYP4A2, (c) CYP4A3, and (d) CYP4A8 mRNA expression levels. (e) Radar map of CYP4A family mRNA changes in kidney tissue. (f) 20-HETE content. (g) COX1 and (h) COX2 mRNA expression levels. (i) PGE_2_ level. (j) LTB_4_ content. (k) BLT1 mRNA expression levels. Data are expressed as mean ± SEM (*n* = 8). ^∗^*P* < 0.05 and ^∗∗^*P* < 0.01 versus the CON group. ^##^*P* < 0.01 versus the AS group. ^§^*P* < 0.05 and ^§§^*P* < 0.01 versus the AS+Alc group. CYP: cytochrome P450; 20-HETE: 20-hydroxystilbenetetraenoic acid; COX: cyclooxygenase; PGE_2_: prostaglandin E_2_; LTB_4_: leukotriene B_4_; BLT1: leukotriene B_4_ receptor 1; CON: control; AS: acute stress; Alc: alcohol.

Compared with the CON and CON+Alc groups, mRNA levels of CYP4A1, CYP4A2, CYP4A3, and CYP4A8 in the AS group were remarkably elevated (*P* < 0.01, Figures 7(a)–7(d)). Subsequent analysis of the expression levels of four CYP4A family enzymes, demonstrated in a radar map, revealed that CYP4A2 was most frequently induced by AS (Figure 7(e)). Furthermore, the 20-HETE content in the AS group was notably higher than that observed in the CON and CON+Alc groups (*P* < 0.01, Figure 7(f)). However, low-dose alcohol significantly reversed these AS-induced alterations (*P* < 0.01).

### 3.8. Effects of Low-Dose Alcohol on the COX/PGE_2_ Metabolic Pathway

As shown in Figures 7(g)–7(i), mRNA expression levels of COX1 and COX2 and PGE_2_ contents in the AS group were not significantly different from those of the CON and CON+Alc groups.

### 3.9. Effects of Low-Dose Alcohol on the LTB_4_/BLT1 Metabolic Pathway

The results shown in Figure 7(j) indicated a significant increase in LTB_4_ levels in kidney tissue of AS rats that was significantly reversed by low-dose alcohol (*P* < 0.01). Furthermore, low-dose alcohol apparently reduced the increase of BLT1 mRNA expression induced by AS (*P* < 0.01, Figure 7(k)).

### 3.10. Correlation Analysis between Activation of CYP4A/20-HETE and LTB_4_/BLT1 Pathways, Oxidative Stress, Proinflammatory Cytokines, and Apoptosis Induced by AS

**Figure 8:**
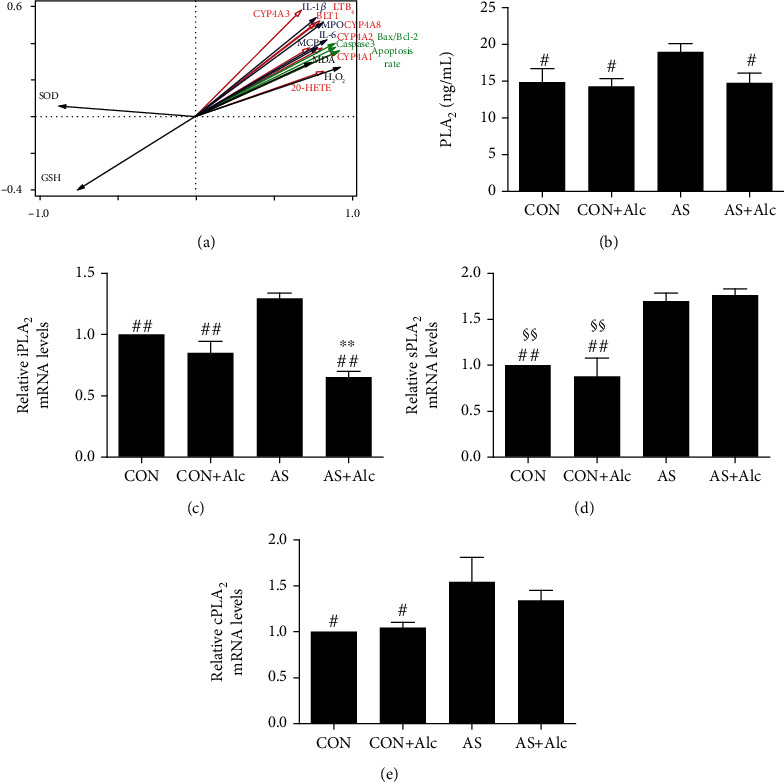
Correlation analysis and effects of low-dose alcohol on phospholipase A_2_ (PLA_2_) activity. (a) Correlation analysis between arachidonic acid metabolism, oxidative stress, proinflammatory cytokines, and apoptosis induced by acute stress. The angle between the arrows represents the correlation. Acute angle: positive correlation. Obtuse angle: negative correlation. Red arrows: related indexes of arachidonic acid metabolism (CYP4A/20-HETE and LTB_4_/BLT1 pathways). Black arrows: oxidative stress index. Blue arrows: proinflammatory cytokines. Green arrows: apoptotic-related indexes. (b) PLA_2_ levels in renal tissues. (c) iPLA_2_, (d) sPLA_2_, and (e) cPLA_2_ mRNA levels. Data are expressed as mean ± SEM (*n* = 8). ^∗∗^*P* < 0.01 versus the CON group. ^#^*P* < 0.05 and ^##^*P* < 0.01 versus the AS group. ^§§^*P* < 0.01 versus the AS+Alc group. iPLA_2_: calcium-independent phospholipase A_2_; sPLA_2_: secreted phospholipase A_2_; cPLA_2_: cytosolic phospholipase A_2_; CYP: cytochrome P450; 20-HETE: 20-hydroxystilbenetetraenoic acid; COX: cyclooxygenase; PGE_2_: prostaglandin E_2_; LTB_4_: leukotriene B_4_; BLT1: leukotriene B_4_ receptor 1; CON: control; AS: acute stress; Alc: alcohol.

Correlation analysis results are shown in Figure 8(a). For activation of the CYP4A/20-HETE metabolic pathway, CYP4A1, CYP4A2, CYP4A3, and CYP4A8 mRNA expression, as well as 20-HETE contents, was positively correlated with levels of MDA, H_2_O_2_, IL-6, IL-1*β*, MPO, MCP-1, Bax/Bcl-2, cleaved caspase 3, and rates of apoptosis and negatively correlated with SOD and GSH activities. Among them, CYP4A1 mRNA expression had the strongest correlation with 20-HETE content, while 20-HETE content had the highest correlation with H_2_O_2_ level. For activation of the LTB_4_/BLT1 pathway, LTB_4_ contents and BLT1 mRNA expression were positively correlated with levels of MDA, H_2_O_2_, IL-6, IL-1*β*, MPO, MCP-1, Bax/Bcl-2, cleaved caspase 3, and apoptosis (especially MPO) and negatively correlated with SOD and GSH activities (especially the latter).

### 3.11. Effects of Low-Dose Alcohol on PLA_2_ Activity

PLA_2_ contents and mRNA levels of iPLA_2_, sPLA_2_, and cPLA_2_ in the AS group were significantly increased compared with those in the CON and CON+Alc groups (Figures 8(b)–8(e)). Interestingly, low-dose alcohol significantly reduced the increase in PLA_2_ and iPLA_2_, but not sPLA_2_ or cPLA_2_.

## 4. Discussion

Alcohol in excess is harmful to health, whereas low doses are beneficial. Indeed, the beneficial effects of low-dose alcohol on organs have been authenticated in numerous studies [27]. The current study has demonstrated that low-dose alcohol (0.05 g/kg), corresponding to 0.25 standard daily drinks (National Institutes of Health definition; a 12-ounce bottle or can of beer containing 5% alcohol, a 5-ounce glass of table wine containing 12% alcohol, or a 1.5-ounce shot of liquor or spirits containing 40% alcohol for a person weighing 70 kg), has a protective effect on AS-induced renal injury, manifested by restoration of renal dysfunction and reduced levels of LEU and BLD. Improvement of histopathological damage provided further evidence for the protective effect of low-dose alcohol against AS-induced renal injury. To our knowledge, this study is the first to explore the protective effect of low-dose alcohol on AS-induced renal injury and the detailed molecular mechanism.

Oxidative stress is considered as a hallmark in AS-induced organ injury [28, 29]. Excessive production of reactive oxygen species (ROS) unbalances the oxidation and antioxidant systems, which triggers oxidative stress [30, 31]. Mechanistically, oxidative stress is implicated in AS-induced renal injury via increased MDA contents and reduced SOD and GSH enzyme activities [5]. MDA, a vital and specific biomarker of oxidative damage, reflects the body's antioxidant potential [32]. Enzymatic SOD and nonenzymatic GSH antioxidants relieve oxidative damage by scavenging ROS (superoxide radicals, hydroxyls, and H_2_O_2_) [33]. In the current study, low-dose alcohol notably suppressed AS-induced MDA and H_2_O_2_ overproduction and elevated SOD activity and GSH concentration. These results indicate that low-dose alcohol has the pharmacological effects of scavenging oxygen free radicals and enhancing the antioxidant defense system. Thus, the antioxidative stress-related pharmacological properties of low-dose alcohol may elicit a protective mechanism against AS-induced renal injury.

Oxidative stress has been implicated in the development of inflammatory processes such as the recruitment of neutrophils [34]. Renal injury is frequently associated with inflammation. Hillegass et al. found that MPO activity was significantly enhanced in inflamed kidney [35]. IL-6 and IL-1*β*, two typical proinflammatory cytokines, play important roles in the inflammatory response [36]. MCP-1, a vital proinflammatory cytokine, is directly involved in the transformation of monocytes into macrophages [37]. Low-dose alcohol reportedly has anti-inflammatory effects [38]. Similarly, we found that low-dose alcohol exerted anti-inflammatory properties in AS-induced renal injury, as evidenced by reduced MPO activity, IL-6 and IL-1*β* concentrations, and MCP levels. Moreover, the observed decrease of LEU content provides further evidence that low-dose alcohol mediated anti-inflammatory effects in the kidney. Therefore, the protective effect of low-dose alcohol against AS-induced renal injury may be partially ascribed to its capability to reduce the production of inflammatory cytokines and weaken the inflammatory response. Notably, the anti-inflammatory properties of low-dose alcohol in acute stress-induced renal injury may be partly related to its antioxidant stress effect.

Apoptosis, an autonomous and orderly form of programmed cell death, has vital biological significance [39]. However, excessive apoptosis can damage a variety of tissues, including the kidney [40]. In the present study, we found that low-dose alcohol alleviated AS-induced apoptosis, as evidenced by a reduction of apoptotic cells. At present, the death receptor-mediated external apoptotic pathway, internal mitochondrial pathway, and endoplasmic reticulum stress pathway are considered the main apoptosis pathways. Our previous study revealed that AS mediates renal cell apoptosis by activating only the endogenous mitochondrial pathway [5]. The proapoptotic protein Bax and antiapoptotic protein Bcl-2 are essential regulators of mitochondrial apoptosis [41]. When mitochondrial dysfunction occurs, Bax is recruited from the cytoplasm to the outer mitochondrial membrane, whereby it is inserted, resulting in oligomerization [42]. Bcl-2, located in the mitochondria, blocks the leakage of apoptotic factors by closing the mitochondrial permeability transition pore. Caspase 3, the executor of the caspase cascade, is activated (cleaved) when the Bax/Bcl-2 ratio is out of balance [43]. We observed that low-dose alcohol decreased Bax/Bcl-2 protein expression ratios and cleaved caspase 3 levels in AS rats. Collectively, the protective effects of low-dose alcohol against AS-induced renal injury may be partly ascribed to its ability to suppress apoptosis.

AA, an essential component of cell membrane lipids, is mainly metabolized by cytochrome P450 enzymes, COX and lipoxygenase (LOX). When the organism is under stress, AA is released from phospholipids as free AA [44], which is metabolized into epoxyeicosatrienoic acid or hydroxyeicosatetraenoic acids by the cytochrome P450 pathway. AA can also be converted into prostaglandins and thromboxanes via the COX pathway. Furthermore, AA generates leukotrienes and lipoxins through the LOX pathway [45]. Nevertheless, in the kidney, hydroxyeicosatetraenoic acids, prostaglandins, and leukotrienes are the main metabolites of AA [46].

The cytochrome P450 pathway is implicated in pivotal renal function and is the primary AA metabolic pathway in the kidney [47]. Notably, the CYP4A family of proteins is highly expressed in the renal cortex and medulla of salt-sensitive rats [48]. At present, four CYP4A subfamily protein subtypes have been found in rat kidney: CYP4A1, CYP4A2, CYP4A3, and CYP4A8 [49]. Moreover, CYP4A1, CYP4A2, and CYP4A3 have been confirmed to possess significant AA *ω*-hydroxylase activity [50]. 20-HETE, the major metabolite produced through *ω*-hydroxylation of AA by CYP4A family proteins, has extensive biological effects, including regulation of renal function [51], constriction of microvessels [52], and raising blood pressure [53]. In addition, 20-HETE can activate ROS production in glomerular podocytes [54]. Suppressing the formation of 20-HETE can alleviate apoptosis, improve albuminuria, and attenuate inflammation [55, 56]. Nilakantan et al. revealed that 20-HETE mediates apoptosis via a superoxide-dependent pathway [57]. In the current study, we observed that mRNA expression of CYP4A1, CYP4A2, CYP4A3, and CYP4A8, as well as 20-HETE contents, was remarkably elevated in AS rats. This phenomenon indicated that AS activated the CYP4A/20-HETE metabolic pathway in the kidney. The radar map shown in Figure 7(e) shows that CYP4A2 was most frequently induced by AS. Interestingly, CYP4A1 had the highest correlation with 20-HETE. These results revealed that the catalytic efficiency of CYP4A1 was higher than that of other CYP4A family proteins, consistent with previous studies [50]. Correlation analysis in this study identified positive correlations between the CYP4A/20-HETE pathway, oxidative stress, inflammation, and apoptosis. Moreover, 20-HETE had the highest correlation with oxidative stress (especially H_2_O_2_); however, low-dose alcohol reversed these AS-induced revisions. Overall, low-dose alcohol could improve AS-induced renal injury by inhibiting the CYP4A/20-HETE metabolic pathway.

COX1 and COX2 are important enzymes in AA metabolism [58]. In the resting state, COX2 is not expressed and COX1 is responsible for regulating the production of PGE_2_ [59]. When the kidney is stimulated, COX2 is highly expressed and mediates massive production of PGE_2_ [60]. Excessive synthesis of PGE_2_ can trigger kidney apoptosis in diabetic rats [61]. Moreover, COX2 induces renal inflammation in diabetic rats by mediating PGE_2_ production [62]. Interestingly, in this study, mRNA expression levels of COX1 and COX2, as well as the content of PGE_2_, were not significantly increased in AS rats. Our findings revealed that the COX/PGE_2_ metabolic pathway was not activated in the kidney of AS rats, a result that may stem from the application of different experimental models.

LTB_4_ is a powerful chemotactic molecule that can mediate inflammation and induce kidney damage [63]. Overexpression of LTB_4_ and BLT1 is an important factor in aggravating inflammation and oxidative stress [64]. Moreover, the LTB_4_-BLT1 axis has been confirmed to induce renal ischemia-reperfusion injury by mediating neutrophil recruitment [65]; it is established that the recruited neutrophils release MPO. In the current study, LTB_4_ levels and BLT1 mRNA expression were significantly increased in AS rats, indicating activation of the LTB_4_/BLT1 pathway. Furthermore, the correlation analysis performed in this study revealed positive correlations between the LTB_4_/BLT1 pathway and oxidative stress, inflammation, and apoptosis. Among them, it had the strongest correlation with inflammation, especially MPO. Importantly, low-dose alcohol significantly reversed these AS-induced alterations. Collectively, low-dose alcohol attenuated AS-induced renal injury, which may be related to the inhibition of the LTB_4_/BLT1 pathway.

PLA_2_, an upstream regulator of the eicosanoid pathway, can liberate free AA from phospholipids [66]. The PLA_2_ superfamily consists of 16 enzymes with reportedly different structures and functions [67]. However, iPLA_2_, sPLA_2_, and cPLA_2_ are all known to be involved in cellular eicosanoid biosynthesis. Our study found that PLA_2_ contents and mRNA levels of iPLA_2_, sPLA_2_, and cPLA_2_ were significantly increased in the AS group. Surprisingly, low-dose alcohol significantly reduced the increases in PLA_2_ and iPLA_2_, but not sPLA_2_ or cPLA_2_. This result may be related to the structure and function of low-dose alcohol. Therefore, inhibition of iPLA_2_ activity may be a vital protective mechanism for low-dose alcohol against AS-induced renal injury; however, the detailed mechanism needs further exploration.

## 5. Conclusions

In conclusion, our results demonstrate that low-dose alcohol protected against AS-induced renal injury by blocking iPLA_2_ activation, inhibiting the CYP4A/20-HETE and LTB_4_/BLT1 pathways, thereby enhancing the antioxidant capacity of the kidney, alleviating inflammation, and improving apoptosis. This study provides useful evidence and a new perspective for the application of low-dose alcohol in the preventive treatment of AS-induced renal injury. Notably, patients with renal disease or stress should abstain from even low-dose alcohol use, as this will adversely affect their clinical treatment process.

## Figures and Tables

**Table 1:** The catalog numbers of all kits.

Kit name	Abbreviations	Catalog number
Malondialdehyde	MDA	A003-1-2
Hydrogen peroxide	H_2_O_2_	A064-1-1
Superoxide dismutase	SOD	A001-3-2
Glutathione	GSH	A006-2-1
Myeloperoxidase	MPO	A044-1-1
Interleukin-6	IL-6	H007-1-2
Interleukin-1*β*	IL-1*β*	H002-1-2
20-Hydroxystilbenetetraenoic acid	20-HETE	JL48233
Prostaglandin E_2_	PGE_2_	H099-1
Leukotriene B_4_	LTB_4_	H552-1
Phospholipase A_2_	PLA_2_	H243-1

**Table 2:** Primer sequence of the relative genes.

Gene	Accession number	Primer sequence (5′-3′)
GAPDH	XM_216453	Forward: AGTGCCAGCCTCGTCTCATA
		Reverse: GATGGTGATGGGTTTCCCGT
CYP4A1	NM-175837	Forward: AGGAGCGAGGAACTGCATTG
		Reverse: CGGAGCTCCACAACGGAATTA
CYP4A2	XM-017593143	Forward: TGTTCAGAGACCCTAGTGATCCA
		Reverse: AGCAGCCATTGCCTTCGTAA
CYP4A3	NM-175760	Forward: AGAGGTCTGCTGCCTGCAATA
		Reverse: TCAGTGGCTGGTCAGAGGTG
CYP4A8	NM-031605	Forward: AGCTGTGGTATCATGAGTGGC
		Reverse: GGAACTGCTGGGTAGCTCTG
COX1	NM-017043	Forward: GTGTACCCACCTTCCGTAGAAC
		Reverse: TAGGATGCTCCTCCTTCAGCA
COX2	NM-017232	Forward: ATTACTGCTGAAGCCCACCC
		Reverse: TGTGATCTGGACGTCAACACG
BLT1	NM-021656	Forward: GGCTAACCTGGAGAGAGCAGT
		Reverse: GCAGATCCACAGACACTGGAG
iPLA_2_	NM-001005560	Forward: AGTTAGGAGTGCTGAGAAGTGC
		Reverse: GGAGTGTCCAGCATATCGCC
sPLA_2_	NM-031598	Forward: CCATACCACCATCCCATCCAAG
		Reverse: CACACCACAATGGCAACCG
cPLA_2_	NM-133551	Forward: GTACCAGAGAACACCTGGGAAG
		Reverse: GGAGTGTCCAGCATATCGCC

## Data Availability

The data used to support the findings of this study are included within the article.
